# Direct and Indirect Cost of Treatment for Chronic Kidney Disease among Patients at the Renal Unit of the University of Nigeria Teaching Hospital, Enugu

**DOI:** 10.4314/ejhs.v34i6.14

**Published:** 2024-11

**Authors:** Ejikeme Benneth Arodiwe, Ijeoma Ogugua Arodiwe, Ijeoma Okoronkwo

**Affiliations:** 1 Department of Medicine, College of Medicine, University of Nigeria, Enugu Campus; 2 Department of Health Administration and Management, Faculty of Health Sciences and Technology, University of Nigeria, Enugu Campus; 3 Department of Paediatrics, College of Medicine, University of Nigeria, Enugu Campus; 4 Department of Nursing, Faculty of Health Sciences and Technology, University of Nigeria, Enugu Campus

**Keywords:** direct cost, indirect cost, chronic kidney disease, end-stage kidney failure, sub-Saharan Africa

## Abstract

**Background:**

Chronic kidney disease (CKD) is a growing global health issue, particularly in low- and middle-income countries. The cost of managing CKD is high, yet there is limited information available regarding this burden in sub-Saharan Africa.

**Methods:**

This descriptive cross-sectional study involved 100 CKD patients at the Renal Unit of a hospital between July and December 2019. Data was collected using a pre-tested, semi-structured questionnaire. Frequencies, means, percentages, standard deviations, medians, and interquartile ranges were used for data analysis. ANOVA and chi-square tests were also employed to assess correlations.

**Results:**

The mean total monthly cost of treatment was 135,199 ± 81,060 Naira ($417 ± 250), with direct and indirect costs averaging 127,565 ± 76,139.8 Naira ($393 ± 235) and 8,309 ± 16,735 Naira ($26 ± 52), respectively. Direct costs accounted for 94% of the total illness cost, with hemodialysis alone representing 86.1% of the total cost. There were no significant differences in treatment costs across socioeconomic classes. Out-of-pocket spending accounted for 88% of healthcare expenses.

**Conclusion:**

The cost of CKD management is high, with hemodialysis comprising the majority of the cost. Most patients pay out of pocket It is recommended that a comprehensive health insurance scheme be implemented, especially to cover hemodialysis treatment.

## Introduction

Chronic kidney disease (CKD) affects approximately 13.4% (11.7%–15.1%) of people worldwide (1). According to the World Health Organization (WHO), CKD is currently the 10th leading cause of death and is projected to become the 5th leading cause by 2040 (2). Research has shown that individuals of African ancestry are at higher risk of developing CKD, with a greater likelihood of progression to end-stage kidney failure (ESKF) (3,4). Factors such as the adoption of Western lifestyles, urbanization, and the APOL1 genetic abnormality contribute to the increasing prevalence of CKD in sub-Saharan Africa (SSA). As a result, the number of CKD patients on the African continent is rising, alongside the dual burden of communicable and non-communicable diseases (5).

Community-based studies in Nigeria have estimated CKD prevalence in adults to range from 19% to 30%, while the pediatric prevalence is approximately 15 per million population (6-8). Hospital-based studies show that end-stage renal disease (ESRD) accounts for 6-12% of medical admissions (9,10). CKD in Nigeria disproportionately affects young individuals in their economically productive years, placing a significant strain on the national economy (11,12).

A previous study at this center demonstrated a high incidence of catastrophic health expenditure among CKD patients, with costs equally distributed across socioeconomic classes (13). The majority of patients financed their treatment out of pocket, using personal savings or borrowing money (13).

The increasing incidence and progression of CKD highlight concerns about the financial strain placed on patients, their families, and the healthcare system. Direct and indirect costs associated with CKD and ESKF are significant, with the most comprehensive data on these costs coming from Europe, the USA, and middle-income countries (14). However, there is a lack of data on the financial burden of CKD in SSA, which hampers effective policy planning and intervention.

This study aims to determine the direct and indirect costs of CKD treatment at the Renal Unit of the University of Nigeria Teaching Hospital and assess whether there are significant differences in treatment costs across various socioeconomic classes.

## Materials and Methods

**Study design and setting**: This was a descriptive cross-sectional study conducted at the Renal Unit of the University of Nigeria Teaching Hospital, Ituku/Ozalla, Enugu. Convenience sampling was used to select 100 patients aged 18 years and above, who had been undergoing treatment for CKD for at least one month. The study was conducted between July and December 2019.

**Sample size estimation**: The sample size was calculated using a known formula (15). The minimum required sample size was 93, which was rounded up to 100 after adjusting for a 10% attrition rate.

**Data collection**: A self-constructed questionnaire was administered by trained assistants to collect data on demographics, household assets, direct and indirect costs, catastrophic expenditure, and coping mechanisms for paying for treatment. The questionnaire was divided into six sections, and its validity was assessed by experts in health economics. A reliability test, using the test-retest method, yielded a reliability score of 0.82.

**Direct and indirect Cost calculation**: Direct costs included consultation fees, laboratory tests, drugs, dialysis, blood transfusions, and transportation. Indirect costs were calculated using the human capital approach, which measures productivity loss based on time spent in treatment. Socioeconomic status (SES) was determined using Principal Component Analysis (PCA) based on household assets.

**Data analysis**: Descriptive statistics (mean, median, interquartile range) were used to summarize the data. Chi-square tests and ANOVA were employed to assess differences in costs across SES quintiles. The significance level was set at p<0.05.

**Ethical approval**: Ethical approval was obtained from the ethics committee of the University of Nigeria Teaching Hospital (UNTH) with approval number NHREC/05/01/2008B-FWA00002458-1RB00002323. All participants provided informed consent

## Results

A total of 100 questionnaires were completed and returned. The majority of participants (52%) were aged 39-58 years, with a mean age of 49.6 ± 16.5 years. Most were male (53%), and 73% were married. The majority of participants (60%) were from lower socioeconomic groups (Q1–Q3), Table 1.

**Table 1:** Socio-economic and demographic characteristics of the study participants (N = 100)

Variables	Frequency (%)
Mean age ±SD. Years	49.6±16.54
Age group	
19-28	13(13)
29-38	17(17)
39-48	24(24)
49-58	28(28)
59-68	9(9)
>69	9(9)
Sex	
Male	53(53)
Female	47(47)
Marital status	
Married	73(73)
Single	19(19)
Widowed	7(7)
Divorced	1(1)
Educational status	
Primary	26(26)
Secondary	21(21)
Tertiary	44(44)
No formal education	9(9)
Source of income	
Government work	23(23)
Private sector	18(18)
Subsistence farming/Artisan	18(18)
Petty trader	22(22)
Unemployed/Pensioner	19(19)
Occupation of Parent or Spouse	
Government work	32(32)
Petty trader	29(29)
Unemployed	20(20)
Private sector	16(16)
Artisan	3(3)
Residence	
Enugu metropolis	44(44)
Other communities in Enugu state	31(31)
Other states	25(25)
Socio-economic status	
Q1(lowest SES, poorest)	14(14)
Q2 very poor	17(17)
Q3 poor	29(29)
Q4 fairly poor	16(16)
Q5(highest SES, least poor)	24(24)

**Direct costs**: Direct costs accounted for 94% of the total treatment costs. Hemodialysis alone made up 86.1% of these costs. The mean monthly expenditure on hemodialysis was 116,375 Naira ($359), with a median of 105,000 Naira ($324). Other direct costs, such as blood transfusions and medications, contributed 22.7% and 19.3% of the total costs, respectively Table 2.

**Table 2:** Direct Cost of CKD treatment per month in Dollar

Cost components	Mean	SD	Median	IQR	% of total cost of treatment
**Direct medical costs**					
Hemodialysis fee	359.2	151	324.1	284.7 – 432.1	86.1
Others**	95.1	70	84.9	46.3 – 131.2	22.8
Cost of drugs	80.9	63.9	58.6	43.2 - 92.6	19.4
Laboratory fee	59	36.9	55.6	40 – 67.9	14.1
Administrative fee	23.4	14.2	21.6	14.5 – 27.8	5.6
Consultation fee	18.3	11.3	15.4	8 – 29.3	4.4
**Direct non-medical cost**					
Transportation	14.3	15.7	6.8	2.8 – 24.7	3.4
**Total Direct cost**	393.7	235	415.6	137 - 566.7	94
**Total Indirect costs**	25.6	51.7	2	1.3 – 25.1	6
**Total costs**	417.3	250.2	438.4	143.5 – 605.3	100

* 324 naira = $1

** Others – cost of blood transfusion, Erythropoietin and Iron injections

**Indirect costs**: The mean indirect cost was 6.1% of the total treatment cost. The highest indirect costs were associated with the time spent on admission and recovery post-discharge. The mean indirect cost related to time spent recovering was 19,569.2 ± 22,617.1 Naira ($60), accounting for 14% of the total medical costs (Tables 3 and 4).

**Table 3:** Indirect cost of Chronic Kidney Disease: Time spent in a month

Time spent (minutes)	Mean	SD	Median	IQR
Transportation	184.5	98.1	120	11.3 – 237.1
Registration	62.1	14.1	60.6	52.1 – 91.9
Consultation	77.6	43.1	66.8	53.2 – 104.7
Laboratory	61.3	16.2	59.5	51.4 – 75.7
Injection room	29.5	6.6	29.4	23.7 – 37.6
Dialysis room	994.5	415	965.2	701.3 – 1207.1
Admission(days)	17.6	18.6	10.4	2 - 29
Back to work after discharge (days)	32.6	37.7	24	14.9 – 39.7

**Table 4:** Indirect cost of CKD per month (Naira)*

Expenses	Mean	SD	Median	IQR	Total direct cost as % total cost of illness
Transport	76.9	40.8	57.9	47.5 – 98.8	0.06
Registration	25.9	5.9	25.3	21.7 – 38.3	0.02
Consultation	32.3	17.9	29.1	22.2 – 43.6	0.02
Laboratory	25.5	6.7	24.8	21.5 – 31.5	0.02
Injection	9.2	6.1	10.6	3.7 – 14.6	0.01
Dialysis	405	181.9	397.2	282.1 – 500	0.30
Admission	13,230	10,989.7	11,200	3450 – 19,028.6	9.8
Back to work	19,569.2	22,617.1	14,400	8925 – 23,800	14.4
**Total**	**8309.1**	**16,734.9**	**637.5**	**429.7 – 8128.1**	**6.1**

* 324 naira = $1

**Socioeconomic status and costs**: There were no significant differences in the direct or indirect costs of treatment across the SES quintiles (p > 0.05). Although the mean direct cost was higher in the least poor (Q5) group (143,000 Naira, $441), compared to the poorest (Q1) group (131,000 Naira, $404), the differences were not statistically significant, Table 5.

**Table 5:** Cost distribution across Socio-economic Class in Dollar

Quintile	Direct cost	Indirect cost
**1st Quintile**		
Mean (SD)	405.6(289)	25.7(40.3)
Median (IQR)	410.5(148 - 545)	1.8(1.1 - 52.9)
**2nd Quintile**		
Mean (SD)	355.4(219.7)	28.1(44.6)
Median (IQR)	435.2(104.9 - 543.2)	13.8(1.6 - 47.7)
**3rd Quintile**		
Mean (SD)	411.9(254.9)	6.6(14.7)
Median (IQR)	417.3(134.3 - 631)	1.7(0.7 - 2.4)
**4th Quintile**		
Mean (SD)	318.2(204.9)	25.1(82.6)
Median (IQR)	264.8(129 – 456.2)	4.7(1.8 – 8.1)
**5th Quintile**		
Mean (SD)	444.2(199)	47.3(59.8)
Median (IQR)	450.3(338.3 – 603.4)	2(1.6 – 105.8)
**Total**		
Mean (SD)	393.7(235)	25.6(51.7)
Median (IQR)	415.6(137 – 566.7)	2(1.3 – 25.1)
*F, df, p value	0.838, 4, 0.505	2.055, 4, 0.093

* F = ANOVA value, df = degree of freedom

**Payment mechanisms**: Out-of-pocket payments without reimbursement were the predominant payment method (88%), with borrowing and personal savings being the most common coping strategies. The least poor (Q5) group had a slightly higher equity ratio for out-of-pocket spending, but the differences were not statistically significant, Table 6.

**Table 6:** Socioeconomic class differences in method of payment

Variable	Q1,(N%)	Q2,(N%)	Q3,(N%)	Q4,(N%)	Q5,(N%)	Total(%)	P value	Q1:Q5
Method of payment							0.660*	
Health Insurance	0	1(1)	1(1)	0	2(2)	4(4)		0
OOP with partial Reimbursement	1(1)	2(2)	3(3)	1(1)	1(1)	8(8)		1
OOP without reimbursement	13(3)	14(14)	25(25)	15(15)	21(21)	88(88)		0.7

X2 13.17, df 16

* p value

## Discussion

This study aimed to quantify the direct and indirect costs of CKD treatment at the University of Nigeria Teaching Hospital, Enugu. The findings highlight the high financial burden faced by CKD patients, particularly due to the cost of hemodialysis, which accounted for the majority of direct medical expenses. Direct costs represented 94% of the total treatment costs, a finding consistent with studies from other low- and middle-income countries (LMICs), where hemodialysis is also a major cost driver (16-19).

The study found no significant differences in treatment costs across socioeconomic classes, despite the fact that most participants (60%) came from lower socioeconomic backgrounds. This suggests inequity in the distribution of healthcare costs, which disproportionately affects poorer patients who are more likely to face catastrophic expenditure.

A significant concern is the lack of financial risk protection for these patients, as 88% of participants paid out of pocket, which can lead to further impoverishment. This highlights the urgent need for policy reforms to ensure that CKD patients are shielded from catastrophic health expenditure. However, the study had several limitations, including the potential for recall bias and the exclusion of certain direct non-medical costs, such as food and lodging. Additionally, the study was conducted before the recent sharp depreciation of the Naira, which may affect the relevance of the cost estimates over time.

The cost of CKD management is prohibitively high, with hemodialysis constituting the largest portion of the expense. Most patients rely on out-of-pocket payments, creating a significant financial burden. Policymakers must prioritize the establishment of financial risk protection schemes, such as comprehensive health insurance, to protect CKD patients from catastrophic expenditure.

In conclusion, this study confirms that the cost of CKD treatment in Nigeria is high and predominantly borne by patients themselves. Hemodialysis, as the primary mode of treatment for many patients, constitutes the largest portion of these costs. There is a need for healthcare policy reform, including the establishment of comprehensive health insurance schemes, to ensure that the financial burden on CKD patients is reduced. Further research should explore the long-term economic impact of CKD and potential strategies for mitigating these costs in sub-Saharan Africa.
